# Arginine metabolism key enzymes affect the prognosis of myelodysplastic syndrome by interfering with macrophage polarization

**DOI:** 10.1002/cam4.6287

**Published:** 2023-06-27

**Authors:** Yang Ou, Yan Yang, Xuefeng Li, Xin Zhang, Lei Zhao, Chenlu Yang, Yu Wu

**Affiliations:** ^1^ Department of Hematology and Hematology Research Institute West China Hospital, Sichuan University Chengdu People's Republic of China

**Keywords:** arginine metabolism, myelodysplastic syndrome, tumor‐associated macrophage

## Abstract

**Introduction:**

Immune factors contribute to the onset of myelodysplastic syndrome (MDS). Arginine metabolism affects tumor‐associated macrophage (TAM) polarization. This study investigated the infiltration of TAMs and effect of arginine metabolism key enzymes on MDS prognosis.

**Methods:**

We used the GEO (Gene Express Omnibus database) dataset “GSE19429” to analyze and compare metabolism‐associated pathways between MDS patients with excess blasts and those without. The markers of TAMs and arginine metabolism key enzymes, including CD68, iNOS, ARG1 and ASS1 were included in this study. A cohort of 79 patients with acute myeloid leukemia or MDS extracted from GenomicScape's online data mining platform was used to analyze the prognostic significance of the mRNA levels. Fifty‐eight patients with primary MDS admitted to Sichuan University's West China Hospital from 2013 to 2017 were evaluated for protein levels. The coexpression of CD68, iNOS, and ARG1 was investigated using an Opal polychromatic immunofluorescence kit.

**Results:**

The “Arginine and proline metabolism” pathways (*p*_adjusted_ = 0.01) were associated with excess blasts in patients with MDS. In the mRNA expression cohort, patients with low NOS2 (or iNOS) and high ARG1, ASS1, and CD68 expression levels had worse prognosis. Patients with high CD68 (*p* = 0.01), high iNOS (*p* < 0.01), low ARG1 (*p* = 0.01), and negative ASS1 (*p* = 0.02) protein expression levels had better prognoses. iNOS and ARG1 were coexpressed with CD68 in MDS patients with or without excess blasts, respectively.

**Conclusions:**

Arginine metabolism may contribute to the prognosis of patients with MDS by affecting TAM polarization.

## 1 INTRODUCTION

Myelodysplastic syndrome (MDS) is characterized by ineffective hematopoiesis and dysplastic morphology and may progress to acute myeloid leukemia (AML) in patients with excessive marrow blasts.^1^ MDS is extremely heterogeneous, implying that the disease course depends on the patient's clinicopathological features (e.g., age and chemotherapy history).

Arginine metabolism plays an important role in many biological pathways, including the urea cycle, TCA cycle, and thyrotropic activities.^2^, ^3^ Arginine is a semi essential amino acid because healthy people can synthesize it from other amino acids. However, human bodies depend on arginine intake to sustain normal functions in pathologic conditions,^4^ for instance, with malignant diseases. In addition, nitric oxide (NO), a critical product of arginine metabolism, can negatively affect tumor proliferation and angiogenesis.^5^, ^6^ As a result, arginine metabolism may be significant in the progression of malignant diseases, and arginine depletion treatments are a promising therapeutic method in many neoplasms.^7^, ^8^ However, when arginine is depleted, arginosuccinate synthetase‐1 (ASS1) is upregulated to sustain the arginine concentration transformed from another amino acid,^9^ which may significantly limit the widespread use of arginine depletion treatments.

In addition to its nutritional significance for malignant cells, arginine metabolism also participates in tumor immunity, especially in MDS. Because chronic inflammatory responses and immune dysfunctions contribute to MDS onset,^10^ arginine metabolism affects tumor‐associated macrophage (TAM) polarization.^11^ Various chemokines can attract monocytes to the tumor microenvironment, where these monocytes participate in tumor immunity. These monocytes then proliferate and differentiate into TAMs in situ. TAMs can affect almost every aspect of tumor cell biology, including cell proliferation, genetic instability, invasion, and metastasis. In activated M1 macrophages, nitric oxide synthase (iNOS) expression is relatively high; thus, arginine is metabolized into citrine and NO, which play an important role in phagocytosis. Increased phagocytic levels were observed in the macrophages of patients with low‐risk MDS, participating in the clearance of precursor granulocytes, and eventually leading to granulocytopenia.^12^ Meanwhile, in M2 macrophages, arginase 1 (ARG‐1) is highly expressed, which metabolizes arginine into urea, polyamine, and ornithine, and participates in tissue repair and immune regulation.^13^ In patients with high‐risk MDS, macrophages promote angiogenesis and disease progression,^14^ similar to the function of M2 macrophages. This study aimed to investigate arginine metabolism key enzymes and their effect on both the polarization of macrophages and MDS prognosis.

## 2 METHODS

### 2.1 Database‐Based transcriptome analysis

The GEO (Gene Express Omnibus database) dataset (GSE19429) was utilized to analyze and compare the metabolism‐associated pathways between MDS patients with excess blasts and those without. The GSE19429 dataset included expression profiles by arrays of CD34^+^ hemopoietic stem cells from 183 patients with MDS (80 patients with excess blasts and 103 patients without).^15^ Differentially expressed genes (DEGs) were obtained after quality control and normalization. Genes with log2‐fold changes of more than 1 and *p* values adjusted for a false discovery rate (FDR) of less than 0.05 were considered significant DEGs. Gene set enrichment analysis (GSEA) and gene set variation analysis (GSVA) were used to elucidate metabolism‐associated pathways. In GSVA, pathways with log2‐fold changes of more than 0.1 and *p* values adjusted for FDR of less than 0.05 were considered significant.

A GenomicScape's online data mining platform was utilized to assess the correlation between the mRNA expression of related genes and patients' survival conditions.^16^ We used a cohort of 79 patients with AML and MDS (GSE12417) to analyze the prognostic significance of these genes, including NOS2 (or iNOS), ARG1, ASS1, and CD68.^17^

### 2.2 Protein expression analysis

From 2013 to 2017, 58 patients with primary MDS admitted to Sichuan University's West China Hospital were included, and their clinical data were collected. Immunohistochemistry (IHC) was used to label the TAMs with anti‐CD68 (catalog no. ab222914, Abcam). The macrophage M1 and M2 subtypes, which were also arginine metabolism key enzymes, were labeled with anti‐iNOS (catalog no. MAB9502, R&D Systems, Minneapolis, United States) and anti‐ARG1 (catalog no. sc166920, Santa Cruz), respectively. Arginine depletion was detected with anti‐ASS1 (catalog no. ab170952, Abcam).

Two independent pathologists blinded to the clinical, pathological, and follow‐up information evaluated the IHC results. The tissues were viewed with an Olympus CCD camera connected to an inverted microscope (Nikon, Eclipse TS 100). The sections were initially viewed under low magnification (1:100) to assess the overall cell distribution pattern, and five nucleated cell‐rich areas were identified. Then, images were captured at a high magnification (1:400) for each “hot spot.” The cytomembrane and cytoplasm were stained brown, with the nuclei unstained, indicating a positive cell. The nucleated and positive cells were individually counted within a field and summed in five regions. The positive cell percentage was calculated for further data analysis. The median positive percentages of different markers were employed as the cut‐off point for dividing patients into low‐ and high‐expression groups. Prognosis was then analyzed using the Kaplan–Meier (KM) regression method.

### 2.3 Coexpression analysis

Formalin‐fixed paraffin‐embedded bone marrows were analyzed using the Opal 4‐Color Manual IHC Kit (catalog no. NEL810001KT, Perkin Elmer) per the manufacturer's instructions. A Nikon A1RMP+ multiphoton confocal microscope was used to capture the fluorescent images. The primary antibodies were anti‐CD68 (catalog no. ab222914, Abcam), anti‐iNOS (catalog no. MAB9502, R&D Systems), and anti‐ARG1 (catalog no. sc166920, Santa Cruz).

### 2.4 Statistical analysis

Statistical analysis was conducted using R language 3.65 (Bell Laboratories; Lucent Technologies). Unpaired Student's *t*‐tests were used to compare the differences between groups, while the Kaplan–Meier (KM) regression method was used for survival analysis. All results are expressed as mean ± SEM. The differences were considered significant at *p* < 0.05. Package limma was utilized to obtain the DEGs.^18^ Package clusterProfiler was used to conduct GSEA analysis,^19^ and package GSVA was used for GSVA analysis.^20^

### 2.5 Ethics statement

This project was approved by an ethical committee of the West China Hospital (Sichuan University). All participants provided written informed consent.

## 3 RESULTS

**FIGURE 1:**
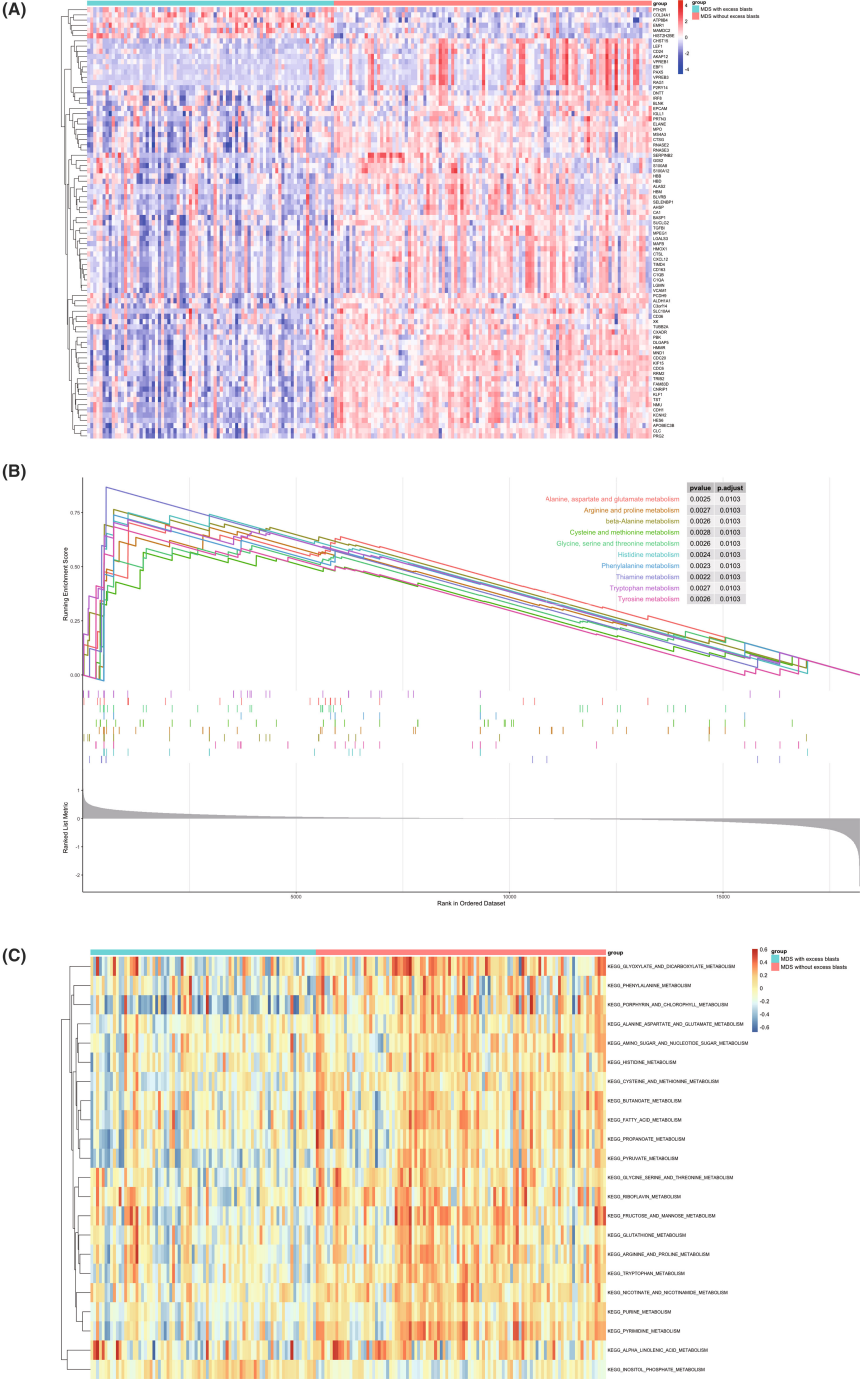
Metabolism‐associated pathways between CD34^+^ hemopoietic stem cells from 80 MDS patients with excess blasts and 103 patients without (GSE19429): (A) The heatmap of DEGs between MDS patients with excess blasts and those without excess blasts; (B) GSEA results about several metabolism pathways of amino acids, including “Arginine and proline metabolism” (*p*_adjusted_ = 0.0103); (C) GSVA also found 54 significant pathways (41 of them were metabolism‐associated) and confirmed that “Arginine and proline metabolism” (*p*_adjusted_ < 0.01) were differentially expressed. MDS, myelodysplastic syndrome; DEGs, differentially expressed genes; GSEA, gene set enrichment analysis; GSVA, gene set variation analysis.

Eighty‐three DEGs were obtained from the GSE19429 between MDS patients with excess blasts and those without (Figure 1A). Arginine metabolism key enzymes were excluded, whereas some genes essential to the immune response were differentially expressed, such as S100A8 and CD163. GSEA enriched several amino acid metabolism pathways, including “arginine and proline metabolism” (*p*_adjusted_ = 0.0103) (Figure 1B). GSVA also found 54 significant pathways (41 of them were metabolism‐associated) and confirmed that “arginine and proline metabolism” (*p*_adjusted_ < 0.01) were differentially expressed between MDS patients with excess blasts and those without (Figure 1C).

**FIGURE 2:**
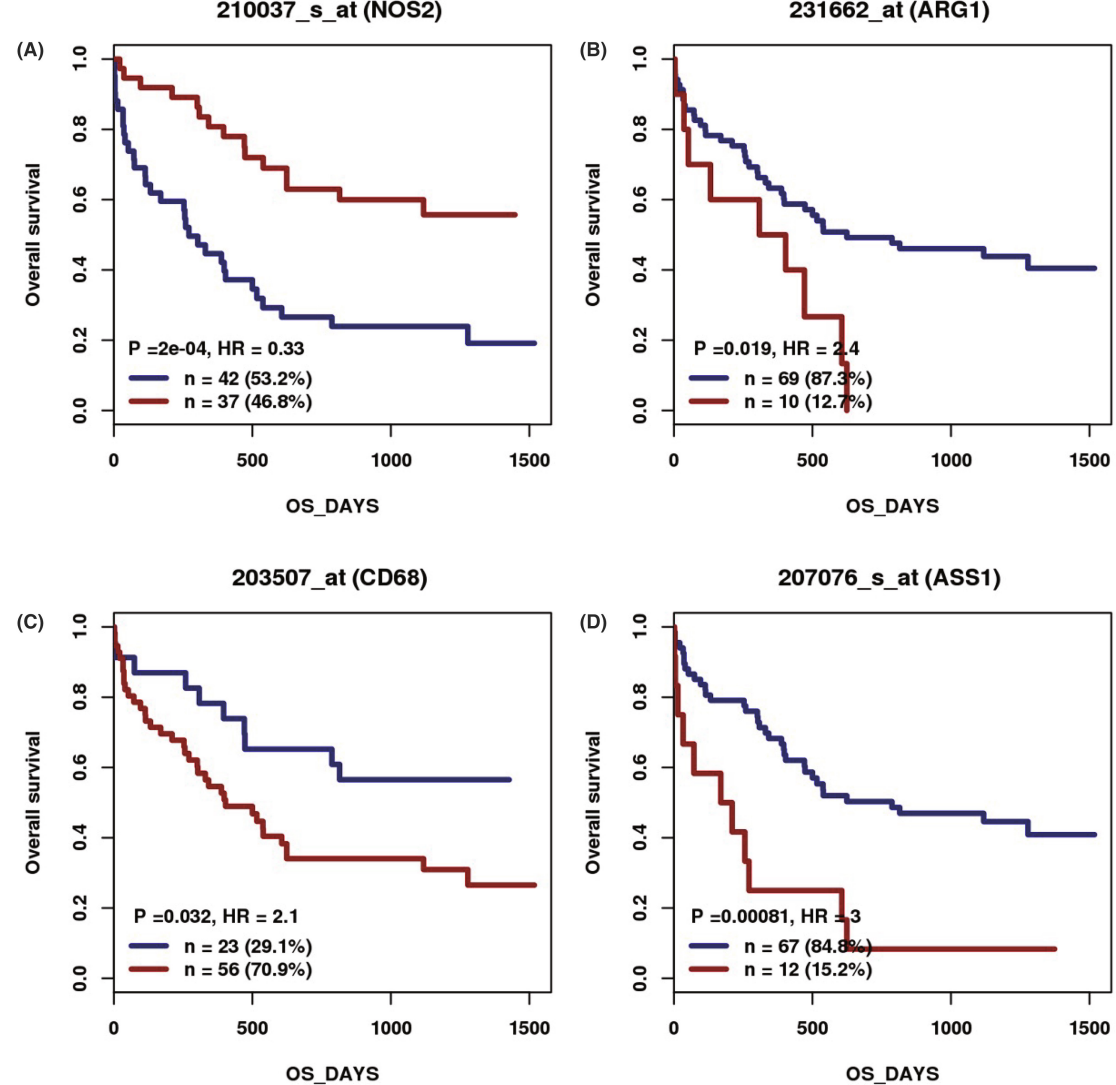
Correlation between mRNA expression of related genes and prognosis in 79 AML and MDS patients (GSE12417) cohort: high NOS2 (or iNOS) expression had a worse prognosis (A, HR = 0.33, *p* < 0.01); however, high ARG1 expression was a negative prognostic indicator (B, HR = 2.4, *p* = 0.019); high CD68 expression had a worse prognosis (C, HR = 2.1, *p* = 0.032), as was high ASS1 expression (D, HR = 3, *p* < 0.01). AML, acute myeloid leukemia; MDS, myelodysplastic syndrome; HR, hazardous rate.

Seventy‐nine patients with AML or MDS were enrolled in the mRNA expression cohort (GSE12417).^17^ In this cohort, patients with a low NOS2 (or iNOS) expression level had a worse prognosis (Figure 2A). The hazardous rate (HR) between high NOS2 and low NOS2 expression levels was 0.33 (*p* < 0.01). In contrast, CD68 had a different prognostic significance compared to NOS2. Patients with high CD68 expression levels had a worse prognosis (Figure 2C, HR = 2.1, *p* = 0.032). High ARG1 (Figure 2B, HR = 2.4, *p* = 0.019) and high ASS1 (Figure 2D, HR = 3, *p* < 0.01) levels were also negative prognostic indicators.

**FIGURE 3:**
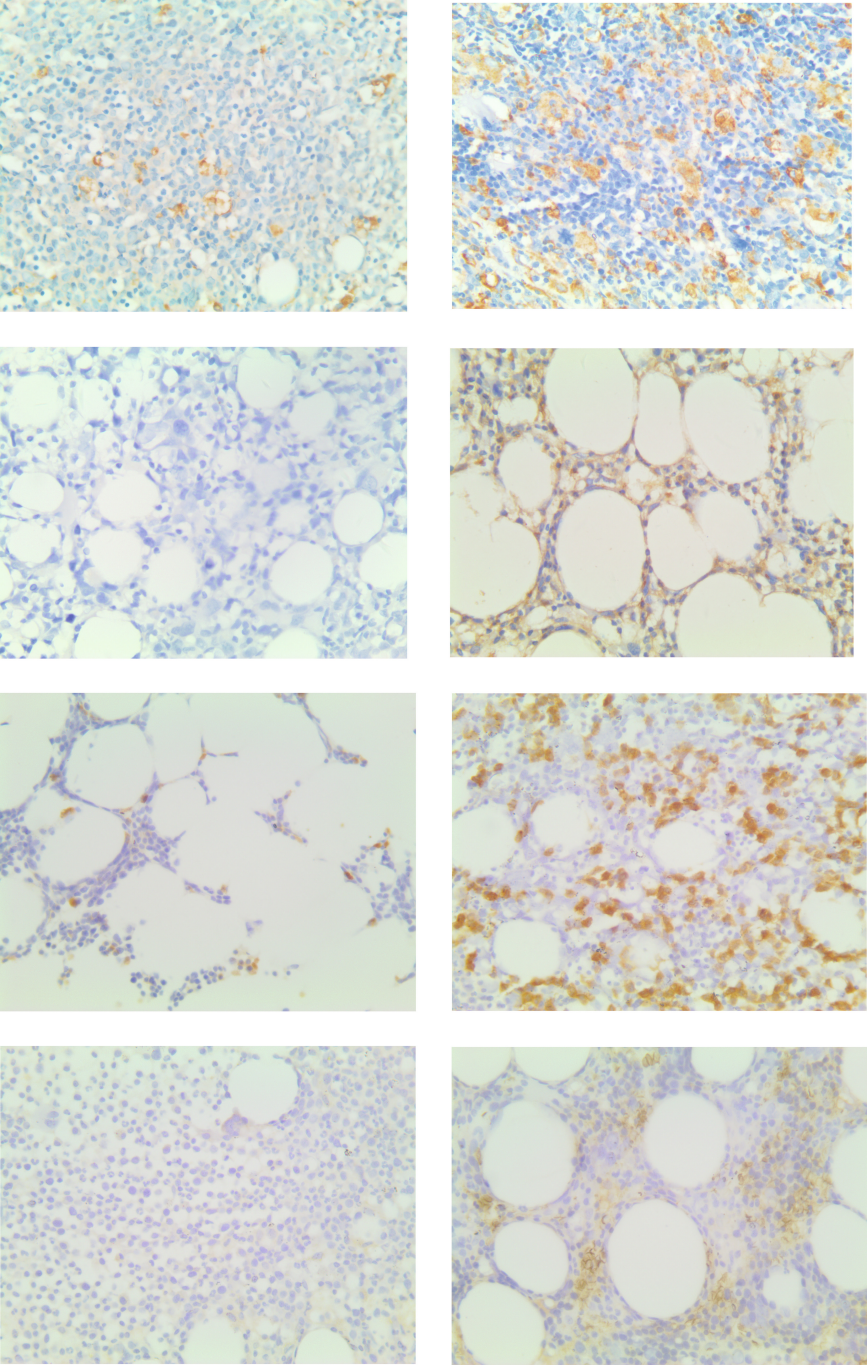
Expressions of CD68, iNOS, ARG1, and ASS1 under high magnification (1:400): low expression is shown on the left, and high expression is shown on the right; CD68, iNOS, ARG1, and ASS1 were listed from top to bottom in order.

CD68 was used to label TAMs, whereas key enzymes in arginine metabolism, including iNOS and ARG1, were harnessed to label M1 and M2 (Figure 3). The average positive expression rates of CD68, iNOS, and ARG1 in all patients with MDS were 0.28, 0.22, and 0.32, respectively. We further analyzed the relationship between CD68, iNOS, and ARG1 expression rates and the clinical characteristics of 58 patients with MDS (Table 1). The gender composition ratio, age, hemoglobin, neutrophil count, platelet count, lactate dehydrogenase, blast cell percentage, and IPSS score did not differ significantly between the low‐expression and high‐expression CD68 or ARG1 group. However, for iNOS, the percentage of blast cells in bone marrow smears in the low‐expression group exceeded that in the high‐expression group (8.83 ± 6.05 vs. 4.81 ± 5.16, *p* = 0.01). The other variables did not show statistically significant differences between the low‐expression and high‐expression iNOS groups.

**TABLE 1:** Clinical characteristics of 58 patients with low or high expression levels of CD68, iNOS, ARG1, and ASS1 in bone marrow immunohistochemistry.

	CD68			iNOS			ARG1			ASS1		
	Low express	High express	*p* value	Low express	High express	*p* value	Low express	High express	*p* value	Negative	Positive	*p* value
Sex (Male/female)	19/10	23/6	0.38	22/7	20/9	0.76	17/11	25/5	0.14	14/8	28/8	0.39
Age	60.05 ± 11.83	58.53 ± 12.21	0.43	58.05 ± 11.83	59.63 ± 14.63	0.73	58.32 ± 11.06	56.26 ± 15.20	0.83	61.73 ± 13.35	57.53 ± 14.18	0.26
Hemoglobin (HGB, g/L)	73.00 ± 24.53	80.38 ± 29.49	0.30	74.31 ± 23.84	79.07 ± 30.33	0.51	75.54 ± 27.02	77.77 ± 27.67	0.76	80.64 ± 28.28	74.28 ± 26.53	0.4
Neutrophil count (Neu × 10^9/L)	1.71 ± 2.68	2.15 ± 2.783	0.54	2.48 ± 3.52	1.39 ± 1.42	0.13	2.07 ± 3.48	1.80 ± 1.79	0.71	2.28 ± 3.04	1.72 ± 2.52	0.48
Plate count (PLT, ×10^9/L)	53.97 ± 65.18	99.66 ± 129.09	0.10	55.93 ± 60.99	97.69 ± 131.80	0.13	78.29 ± 80.66	75.43 ± 123.18	0.92	74.36 ± 74.93	78.31 ± 119.25	0.88
Lactate dehydrogenase (LDH, IU/L)	286.72 ± 140.02	296.28 ± 256.18	0.86	340.66 ± 260.63	242.34 ± 111.11	0.07	282.68 ± 267.47	299.73 ± 124.85	0.76	295.50 ± 232.18	289.06 ± 189.38	0.91
Blast percentage (%)	7.01 ± 5.72	6.63 ± 6.24	0.81	8.83 ± 6.05	4.81 ± 5.16	0.01	5.73 ± 5.75	7.85 ± 6.01	0.18	5.54 ± 6.00	7.61 ± 5.84	0.2
IPSS scoring	1.36 ± 0.73	1.33 ± 0.83	0.87	1.69 ± 0.77	1.00 ± 0.61	<0.01	1.34 ± 0.87	1.35 ± 0.68	0.96	1.25 ± 0.80	1.40 ± 0.76	<0.01

**FIGURE 4:**
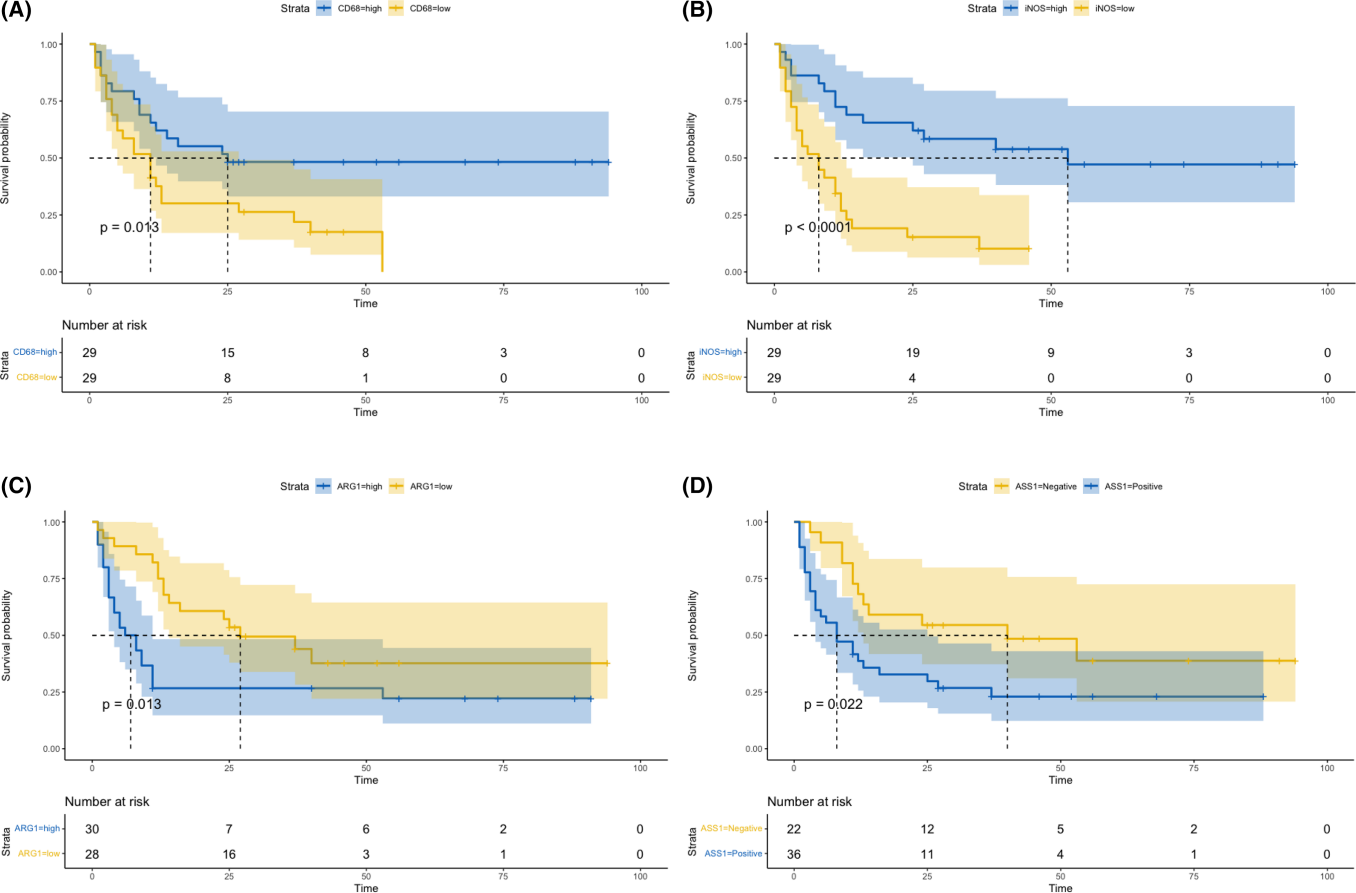
The survival plots of the expression levels of different markers stained by the immunohistochemical method: (A) The KM survival curve of patients with high CD68 and low CD68 expression levels (*p* = 0.01); (B) The KM survival curve of patients with high iNOS and low iNOS expression levels (*p* < 0.01); (C) The KM survival curve of patients with high ARG‐1 and low ARG‐1 expression levels (*p* = 0.01); (D) The KM survival curve of patients with high ASS‐1 and low ASS‐1 expression levels (*p* = 0.02). KM, Kaplan–Meier regression method.

According to the univariate cox proportional hazards model, the presence of excessive blasts, low hemoglobin levels, high IPSS scores, as well as low CD68 levels, low iNOS levels and high ARG1 levels, high ASS1 levels were bad prognostic factors (Table S1). And in kaplan–meier survival analysis, Patients with high CD68 expression levels had a higher survival probability and better prognosis than those with low CD68 expression levels (Figure 4A *p* = 0.01). Patients with high CD68 expression levels had a median survival time of 25 months, whereas those with low CD68 expression levels had only an 11‐month median survival time. Patients with high iNOS expression levels were also more likely to have a better prognosis than those with low iNOS expression levels (Figure 4B *p* < 0.01, median survival time 53 vs. 8 months). The prognostic significance of ARG1 differed from that of CD68 and iNOS. Patients with high ARG1 expression levels had a shorter median survival time (7 vs. 27 months) and a lower survival probability than those with low ARG1 expression levels (Figure 4C *p* = 0.01). As an indicator of arginine depletion, ASS1 had a similar prognostic significance to ARG1. Negative ASS1 expression correlated with a longer median survival time (8 vs. 40 months) and a better prognosis (Figure 4D *p* = 0.02).

**FIGURE 5:**
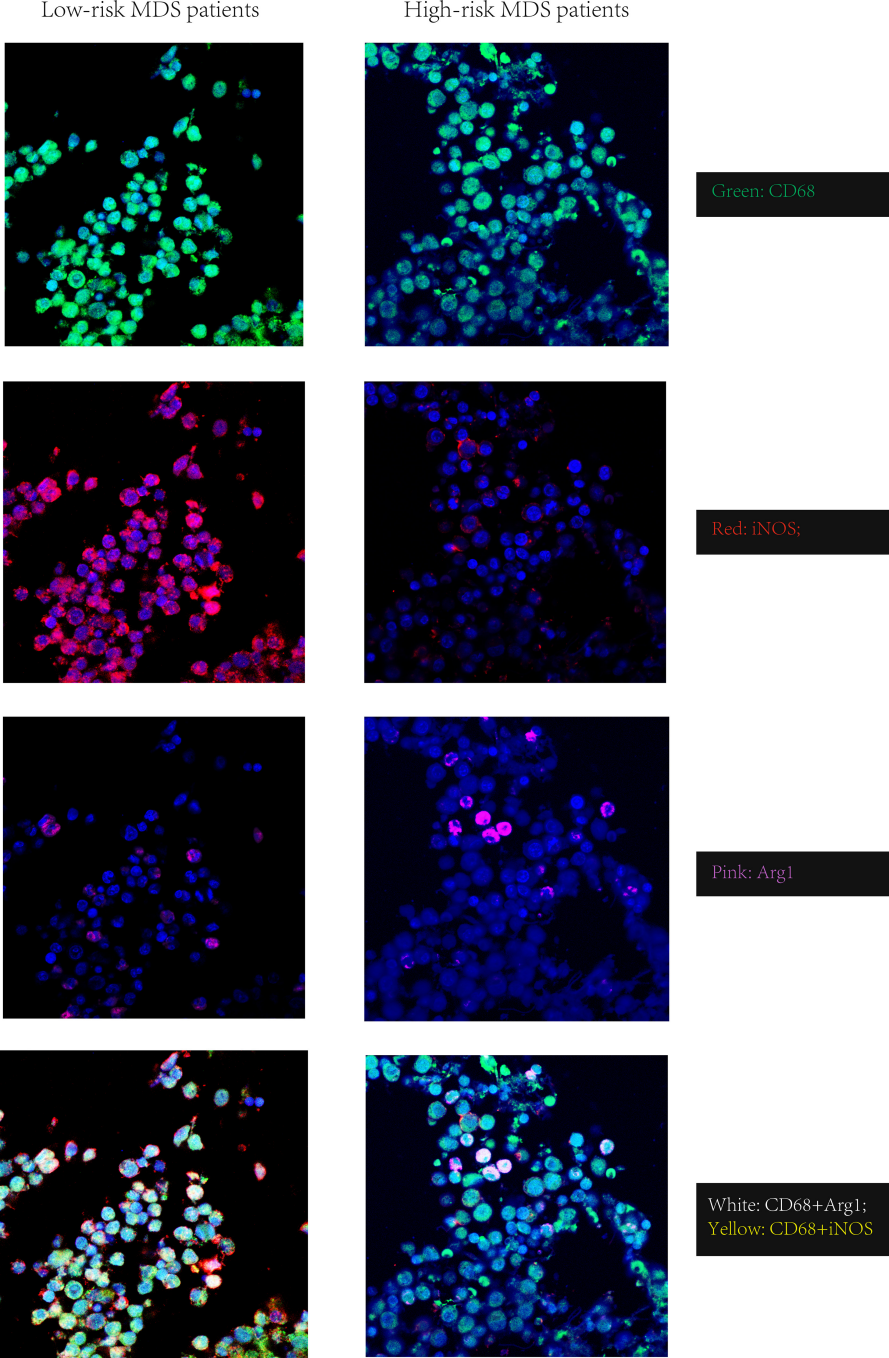
Coexpression of iNOS with CD68 and ARG1 with CD68 using polychromatic immunofluorescence (×200): iNOS was coexpressed with CD68 in low‐risk MDS patients (left), whereas ARG1 was coexpressed with CD68 in high‐risk MDS patients (right).

Multicolor immunofluorescence showed that iNOS was coexpressed with CD68 in patients with low‐risk MDS (Figure 5, left), whereas ARG1 was coexpressed with CD68 in patients with high‐risk MDS (Figure 5, right), indicating that arginine metabolism might be important in TAM polarization.

## 4 DISCUSSION

Many metabolic pathways can influence the direction of the macrophage polarization process.^21^ The fatty acid oxidation is preferentially activated in M2 macrophages, and inhibiting this process halts M2 activation.^22^ To obtain energy, M1 and M2 macrophages preferentially use glycolysis and oxidative phosphorylation, respectively.^23^ In the late phase of M1 macrophage polarization, lactate production by glycolysis can increase histone lactylation, induce homeostatic genes involved in wound healing, including Arg1, and promote homeostasis.^24^ This provided novel insight into the role of metabolism in macrophage polarization.

However, among these metabolic pathways, arginine metabolism is crucial. The expression of different enzymes related to arginine metabolism also varies remarkably, thus affecting the activation of M1‐ and M2‐type macrophages.^21^ In M1 macrophages, iNOS is highly expressed, and arginine is metabolized to NO and citrulline, playing the role of immune stimulation and inhibiting tumor cell proliferation.^25^ In contrast, ARG1 is highly expressed in M2 macrophages and metabolizes arginine into urea, polyamine, and ornithine, which are involved in macrophage repair and play immunosuppressive roles in assisting tumor invasion.^26^, ^27^ In addition, ASS1 expression enables macrophages to synthesize arginine from imported citrulline to sustain NO output when extracellular arginine is depleted, thereby maintaining macrophages' immune functions.^28^ The coexpression of CD68 with iNOS or ARG1 in patients with low‐risk MDS (Figure 5, left) or patients with high‐risk MDS (Figure 5, right), respectively, supported these inferences.

Many studies on hematologic malignancies have reported that TAM invasion in the bone marrow negatively correlates with the prognosis.^29^ Other researchers also found that MDS macrophages exhibited more M2‐related characteristics,^30^ especially in patients with high‐risk MDS,^31^ corroborating the conclusion that patients with high CD68 expression levels had a worse prognosis in the mRNA expression cohort (GSE12417)^17^ (Figure 2C, HR = 2.1, *p* = 0.032). However, our IHC results showed a different conclusion. In our research, MDS patients with high CD68 expression levels had a higher survival probability and better prognosis than patients with low CD68 expression levels (Figure 4A, *p* = 0.01). This may result from the fact that MDS is more benign than other hematologic malignancies, and GSE12417's patients are mainly AML and MDS with excess blasts, in which M2 type macrophages play a more important role compared to relatively lower risks and less M2‐related characteristics in our MDS patients.

We also found that patients with high iNOS expression had a better prognosis than those with negative expression (Figure 4B, *p* < 0.01), whereas the prognostic significance of ARG1 opposes that of CD68 and iNOS, and patients with high ARG1 expression had a lower survival probability than those with low ARG1 expression (Figure 4C, *p* = 0.01). This corroborates the mRNA expression cohort (GSE12417)'s results and the role of iNOS and ARG‐1 in arginine metabolism and macrophage polarization.

ASS1 is an indicator of arginine depletion, and extracellular arginine depletion causes the polarization of macrophages toward the M2 type,^32^ which plays an immunosuppressive role in assisting tumor invasion.^26^, ^27^ Accordingly, our results showed that negative ASS1 expression had a longer median survival time (8 vs. 40 months) and better prognosis (Figure 4D, *p* = 0.02).

Recent advances have focused on developing novel drugs that target immunological pathways for MDS.^33^ Targeting macrophages, Hu5F9‐G4, a humanized anti‐CD47 blocking antibody, is undergoing Phase I MDS clinical trials.^34^ This antibody can block the CD47–SIRPα interaction, which plays an important role in the immune tolerance of macrophages. Previous research also identified that targeting IRF7‐SAPK/JNK pathway to induce M1 characteristics in TAMs could be a potential target for macrophage based immuno‐therapy strategy against leukemia.^35^ Surveillance of arginine metabolism may contribute to predicting and assessing such treatment. In addition, some studies have found that cells from most patients with AML are deficient in the enzyme ASS1, which is critical for arginine synthesis.^36^ This means that AML and high‐risk MDS are arginine auxotrophic tumors for which l‐arginine is essential for survival and progression. Thus, the appropriate deprivation of l‐arginine may be an effective therapy for AML and high‐risk MDS.

## 5 CONCLUSIONS

The pathway of “Arginine and proline metabolism” plays an important role in the excess blasts of MDS patients. In the mRNA expression cohort, patients with low NOS2 expression and high ARG1, ASS1 and CD68 expression had a worse prognosis. Patients with a high protein expression level of CD68, high level of iNOS, low level of ARG1, and negative ASS1 had a better prognosis, and iNOS and ARG1 are co‐expressed with CD68 in MDS patients with or without excess blasts, respectively.

## AUTHOR CONTRIBUTIONS

**Yang Ou:** Conceptualization (equal); data curation (equal); formal analysis (equal); methodology (equal); software (equal); visualization (equal); writing – original draft (equal). **Yan Yang:** Conceptualization (equal); investigation (equal); methodology (equal). **Xuefeng Li:** Software (equal); validation (equal); visualization (equal). **Xin Zhang:** Resources (equal); software (equal). **Lei Zhao:** Software (equal); validation (equal). **Chenlu Yang:** Methodology (equal); supervision (equal). **Yu Wu:** Conceptualization (lead); funding acquisition (lead); investigation (lead); project administration (lead); supervision (lead); writing – review and editing (lead).

## FUNDING INFORMATION

This work was supported by grants from “1·3·5 project for disciplines of excellence–Clinical Research Incubation Project, West China Hospital, Sichuan University” and the Sichuan Provincial Academic and Technical Support Funding Project (2022YFS0191).

## CONFLICT OF INTEREST STATEMENT

The authors declare no competing financial interests.

## Supporting information


Table S1.

## Data Availability

All data generated or analyzed during this study are included in this published article and its supplementary information files.
