# Supplementary material for: Arginine metabolism key enzymes affect the prognosis of myelodysplastic syndrome by interfering with macrophage polarization
Source: Cancer Med. 2023 Jun 27;12(15):16444–54. doi: 10.1002/cam4.6287 (PMC10469818; doi:10.1002/cam4.6287)
Supplement: Supplementary file 1 — Table S1. [file CAM4-12-16444-s001.docx]

Supplementary Table 1. Univariate Cox proportional hazards model of the clinical characteristics and immunohistochemistry markers of the 58 patients in immunohistochemistry cohort.

|  | Hazard Ratio (HR) | P value |
| --- | --- | --- |
| Age | 1.01 (0.9861, 1.035) | 0.4 |
| Hemoglobin (HGB, g/L) | 0.988 (0.9757,1) | 0.05 |
| Neutrophil Count (Neu ×10^9/L) | 1.057 (0.9639, 1.158) | 0.2 |
| Plate Count (PLT, ×10^9/L) | 0.994 (0.989, 0.999) | 0.02 |
| lactate dehydrogenase (LDH, IU/L) | 1.001 (0.9997, 1.002) | 0.1 |
| Blast percentage (%) | 1.094 (1.036, 1.155) | <0.01 |
| IPSS scoring | 1.534 (1.062, 2.216) | 0.02 |
| CD68 expression level | 0.4435 (0.2304, 0.8535) | 0.01 |
| iNOS expression level | 0.2674 (0.1332, 0.5368) | <0.01 |
| ARG1 expression level | 2.265 (1.179, 4.351) | 0.01 |
| ASS1 expression level | 2.193 (1.106, 4.347) | 0.02 |
